# Predicting vaccine effectiveness against invasive pneumococcal disease in children using immunogenicity data

**DOI:** 10.1038/s41541-022-00538-1

**Published:** 2022-11-07

**Authors:** Josiah Ryman, Jessica Weaver, Tianyan Hu, Daniel M. Weinberger, Ka Lai Yee, Jeffrey R. Sachs

**Affiliations:** 1 Quantitative Pharmacology and Pharmacometrics, Merck & Co., Inc, Rahway, NJ USA; 2 Center for Observational and Real-World Evidence, Merck & Co., Inc., Rahway, NJ USA; 3 Department of Epidemiology of Microbial Diseases, Yale School of Public Health, New Haven, CT USA

**Keywords:** Conjugate vaccines, Bacterial infection

## Abstract

The strength of the immune response, as measured by antibody concentrations, varies between pneumococcal conjugate vaccines (PCVs). Linking immunogenicity and effectiveness is necessary to assess whether changes in immune response from currently recommended PCVs to next-generation vaccines could impact effectiveness. Simulated reverse cumulative distribution curves were generated using published serotype-specific IgG concentrations with placebo or PCV7. This was combined with the published estimates of serotype-specific vaccine effectiveness of PCV7 against invasive pneumococcal disease to estimate the protective antibody concentration for each serotype in PCV7. Then, based on the published serotype-specific IgG concentrations in PCV13 recipients, reverse cumulative distribution curves were generated for the serotypes shared between PCV13 and PCV7. These estimated protective antibody concentration values were then used to predict the vaccine effectiveness of PCV13. The results were compared to published aggregate values for vaccine effectiveness. The aggregate median predicted vaccine effectiveness values were similar to previously reported observed values for the United Kingdom (93% versus 90%), Australia (71% versus 70%), and Germany (91% versus 90%). These results demonstrate that IgG concentrations of next-generation PCVs can be used to generate reliable estimates of vaccine effectiveness for serotypes shared with established PCVs.

## Introduction

*Streptococcus pneumoniae* is a major cause of meningitis, bacteremia, and pneumonia worldwide and is responsible for over 300,000 deaths annually in children under the age of 5^1^. Although pneumococcal conjugate vaccines (PCVs) have substantially reduced the burden of invasive pneumococcal disease (IPD) in children and adults, breakthrough disease and serotype replacement (i.e., an increase in the frequency of non-vaccine serotypes) are ongoing concerns^1–3^.

The first PCV approved for use in children targeted seven serotypes (PCV7), and subsequent PCVs expanded the valency to 10 (PCV10) and 13 (PCV13) serotypes^4^. Higher valency PCVs are being developed to target an even larger number of serotypes^5^. Ideally, the efficacy of these new PCVs would be evaluated in randomized controlled trials (RCTs). However, it is not feasible to conduct RCTs for several reasons: if populations are already using PCVs, the number of events would be too small for meaningful evaluations and comparisons between products. Additionally, placebo-controlled trials are not considered ethical when an effective vaccine is available^6^. Therefore, new PCVs, starting with PCV10, have been evaluated via their immunogenicity and approved by the FDA if they elicit an immune response that is non-inferior to existing PCVs. For pediatric vaccines, the primary outcome used for evaluating new PCVs is the concentration of immunoglobulin G (IgG) that targets the pneumococcal capsule. Because licensure decisions are based on IgG concentrations, this is the primary basis of comparison between new and existing PCVs until vaccine effectiveness (VE) studies are performed (typically several years after the introduction of the new PCVs). Inevitably, technical advisory groups and decision makers will try to interpret differences in immunogenicity between vaccines and make inferences about effectiveness. For instance, comparing PCV13 and PCV7, there was a weaker IgG response for all of the PCV7 serotypes following the primary doses of PCV13, and weaker response after the booster dose for all of the PCV7 serotypes except 19F^7^.

While IgG concentrations are the standard basis of comparison, decision-makers are interested in the implications of these differences for VE. Differences in immunogenicity between PCVs can be misinterpreted. There is therefore a need for a framework that contextualizes differences in PCVs and attempts to express any differences in terms of VE.

The standard practice in the pneumococcal community has been to interpret an IgG concentration of 0.35 µg/mL as a correlate of protection against IPD, with that same correlate of protection used for all serotypes^8^. This is based on a method by Siber et al. that determines the protective concentration of IgG for the PCVs, based on the vaccine efficacy measured in clinical trials^9^. The correlate of protection actually varies by serotype and population^9,10^, and this variation needs to be taken into account when projecting VE based on differences in immunogenicity.

In the present analysis, we leverage and advance Siber’s method to calculate protective concentrations (C_p_) using real-world effectiveness data and then apply Siber’s method “in reverse” to show that a known C_p_ can be used to predict the effectiveness of higher valency vaccines, and that this can be done in a serotype-specific manner using summary-level data. The objective of this work was to assess the utility of this method for predicting the serotype-specific effectiveness of next-generation PCVs. The assessment is achieved by predicting PCV13’s effectiveness against the PCV7 serotypes and then comparing the predicted effectiveness values with corresponding published values.

## Results

### Serotype-specific protective antibody concentration

Serotype-specific C_p_ for the PCV7 serotypes were calculated using the source data from each country (Table 1) as input to the calculations described under “Methods” below. Median values ranged from 0.08 (serotype 6B) to 1.27 (serotype 19 F) µg/mL in the United Kingdom, from 0.64 (serotype 23 F) to 6.08 (serotype 19 F) µg/mL in Australia, and from 0.08 (serotype 6B) to 2.96 (serotype 4) µg/mL in Germany.

**Table 1:** Predicted serotype-specific protective antibody concentration for the PCV7 serotypes in PCV13.

Serotype	Protective antibody concentration Median (95% CI), µg/mL		
	United Kingdom	Australia	Germany
4	0.40 (0.18–1.29)	0.75 (0.44–1.52)	2.96 (0.46-∞^a^)
6B	0.08 (0.02–2.28)	1.38 (0.38-∞^a^)	0.08 (0.02–0.75)
9 V	0.51 (0.17-∞^a^)	0.93 (0.55–1.90)	0.87 (0.27-∞^a^)
14	0.63 (0.22–1.58)	1.92 (0.57-∞^a^)	0.63 (0.22–2.82)
18 C	0.15 (0.05–0.43)	0.84 (0.38–3.69)	1.13 (0.37-∞^a^)
19 F	1.27 (0.52–5.19)	6.08 (1.05-∞^a^)	1.50 (0.47-∞^a^)
23 F	0.20 (0.06–1.87)	0.64 (0.20-∞^a^)	0.34 (0.08-∞^a^)

*CI* confidence interval.

^a^The infinite estimates correspond to serotype-specific effectiveness inputs with lower bounds that are negative, or very close to being negative. In cases where the lower bound of the VE is negative, there would be simulations that would sample and input these negative effectiveness values into the calculation. When this happens a C_p_ cannot be estimated, and an infinite value is produced.

### Serotype-specific vaccine effectiveness

Serotype-specific VE values were predicted for each of the seven serotypes shared between PCV7 and PCV13 using the immunogenicity data from each country (United Kingdom, Australia, and Germany) along with the C_p_ values calculated in the first step (Table 2).

**Table 2:** Predicted serotype-specific vaccine effectiveness for the PCV7 serotypes in PCV13.

Serotype	Vaccine effectiveness Median (95% CI)		
	United Kingdom	Australia	Germany
4	97% (61–100)	87% (59–97)	35% (2–97)
6B	86% (3–100)	70% (15–94)	94% (61–100)
9 V	75% (1–98)	60% (26–82)	80% (1–99)
14	97% (86–100)	86% (26–99)	97% (68–100)
18 C	99% (86–100)	80% (11–98)	74% (3–97)
19 F	91% (50–99)	12% (2–89)	56% (4–91)
23 F	87% (22–99)	67% (3–93)	88% (6–99)

*CI* confidence interval.

### Aggregate vaccine effectiveness

No serotype-specific values of VE for PCV13 were available for comparison, so previously reported aggregate values (for the types in PCV7) were used as a standard. The predicted aggregate VE values are shown in Table 3. The median values for each prediction were close to the previously observed values for the United Kingdom (93% predicted versus 90% reported^10^), Australia (71% versus 70%^11^), and Germany (91% versus 90%^12^).

**Table 3:** Predicted versus observed aggregate vaccine effectiveness for the PCV7 serotypes in PCV13.

	Vaccine effectiveness Median (95% CI)		
	United Kingdom	Australia	Germany
Predicted	93% (20–100)	71% (3–97)	91% (5–100)
Observed	90% (34–98)^a^	70% (−8–92)^b^	90% (57–98)^c^

*CI* confidence interval.

^a^Data are from Andrews et al.^10^.

^b^Data are from Jayasinghe et al.^11^.

^c^Data are from Weinberger et al.^12^.

Lower bounds of the confidence intervals deviated more widely from the observations in both the United Kingdom and Germany. The deviation of the lower bound is due to the method for VE prediction capturing more variability in VE overall compared to what was observed. The increased variability in effectiveness is reflected at both bounds of the prediction, but because the upper bound of the prediction cannot exceed 100%, the deviation is not as profound at the upper bound. The deviation seen on the lower bound of the aggregate prediction is due to the high variability in the serotype-specific VE input data, in some cases varying from negative values to 100% (Table 4). In cases where effectiveness input values were highly variable, the protective concentration estimation would be highly variable which then leads to a highly variable effectiveness prediction. For Australia, the predicted lower bound of the confidence interval was higher than the reported value of −8%. The reported −8% may not be realistic since this suggests that PCV13 increases IPD. The results are most likely due to the variability resulting from a relatively small number of cases on one or more of the included serotypes. Furthermore, to predict a negative VE with our method, the placebo reverse cumulative distribution curve (RCDC) must have a higher percentage of subjects that meet or exceed an IgG concentration compared to the PCV13 treated arm, which was not observed for any serotype in PCV13. Based on the observed placebo and PCV13 geometric mean concentrations (GMCs) and distributions, the 3% lower bound estimate may be more realistic.

**Table 4:** Summary-level input data.

Serotype		PCV7 VE	Serotype-specific antibody concentration Geometric mean (95% CI), µg/mL		
			Placebo	PCV7	PCV13
United Kingdom	Sources:	Andrews et al.^10^	Siber et al.^9^	Findlow et al.^19^	Ladhani et al.^20^
4		97% (65–100)	0.03 (0.02–0.03)	1.61 (1.40–1.84)	1.55 (1.41–1.70)
6B		58% (3–82)	0.08 (0.07–0.09)	0.20 (0.16–0.25)	0.32 (0.29–0.36)
9 V		70% (−25–93)	0.05 (0.05–0.06)	0.87 (0.73–1.04)	0.93 (0.83–1.04)
14		98% (88–100)	0.05 (0.04–0.06)	5.03 (4.17–6.07)	5.28 (4.54–6.13)
18 C		96% (81–99)	0.04 (0.04–0.05)	1.11 (0.91–1.35)	1.19 (1.06–1.34)
19 F		75% (37–90)	0.11 (0.09–0.13)	2.67 (2.19–3.26)	4.57 (4.04–5.16)
23 F		78% (23–94)	0.05 (0.04–0.06)	0.55 (0.44–0.68)	0.69 (0.60–0.79)
Australia	Sources:	Jayasinghe et al.^11^	Siber et al.^9^	Siber et al.^9a^	NCT00444457^22^^a^
4		72% (49–85)	0.03 (0.02–0.03)	1.36 (1.20–1.56)	1.75 (1.63–1.88)
6B		75% (1–94)	0.08 (0.07–0.09)	3.34 (2.75–4.05)	2.54 (2.27–2.85)
9 V		72% (49–85)	0.05 (0.05–0.06)	1.60 (1.41–1.83)	1.11 (1.04–1.19)
14		82% (−76–98)	0.05 (0.04–0.06)	4.68 (4.07–5.40)	5.18 (4.72–5.69)
18 C		81% (32–94)	0.04 (0.04–0.05)	1.96 (1.71–2.25)	1.48 (1.38–1.58)
19 F		7% (−100–72)	0.11 (0.09–0.13)	1.44 (1.26–1.65)	2.59 (2.40–2.78)
23 F		76% (−3–95)	0.05 (0.04–0.06)	1.44 (1.22–1.70)	1.03 (0.94–1.14)
Germany	Sources:	van der Linden et al.^18^	Siber et al.^9^	NCT00366340^21^	NCT00366340^21^
4		51% (−1088–100)	0.03 (0.02–0.03)	2.99 (2.68–3.33)	2.18 (1.98–2.40)
6B		97% (72–100)	0.08 (0.07–0.09)	1.49 (1.27–1.75)	0.98 (0.84–1.14)
9 V		83% (−158–100)	0.05 (0.05–0.06)	1.96 (1.77–2.17)	1.65 (1.51–1.80)
14		97% (71–100)	0.05 (0.04–0.06)	4.61 (4.07–5.23)	4.14 (3.68–4.66)
18 C		79% (−137–100)	0.04 (0.04–0.05)	2.25 (2.04–2.49)	1.94 (1.76–2.14)
19 F		73% (−44–97)	0.11 (0.09–0.13)	2.86 (2.53–3.24)	1.73 (1.56–1.92)
23 F		89% (−23–100)	0.05 (0.04–0.06)	1.44 (1.25–1.65)	1.26 (1.11–1.43)

*CI* confidence interval, *PCV* pneumococcal conjugate vaccine, *VE* vaccine effectiveness.

^a^One-month post-primary vaccination for subjects given either PCV7 or PCV13 in a 3 + 0 regimen was unavailable in the Australian population. Trial data from the United States were used here because the primary infant dosing regimen is the same (3 + 0) and the populations are assumed to have similar immune responses to PCV7 and PCV13.

To evaluate the performance of predicted VE and the importance of C_p_ values, we ran simulations using the method described using the commonly accepted 0.35 µg/mL protective threshold (for every serotype). The results in Table 1 show C_p_ values ranging from 0.08 to 6.08. The changes in the resulting VE (Table 5; relative to values estimated using the fixed value of C_p_ = 0.35) show that proper, serotype-specific C_p_ estimation is needed to estimate serotype-specific VE: in cases where the protective thresholds are substantially different than 0.35, the resulting predicted VE values are also substantially different (Table 5). Estimation of serotype-specific C_p_ also results in better alignment between predicted and reported aggregate (incidence rate-weighted) VE (Table 5).

**Table 5:** Predicted serotype-specific vaccine effectiveness for the PCV7 serotypes in PCV13: Estimations using serotype-specific protective threshold compared to the estimations using pan-serotype 0.35 µg/mL correlate of protection.

Serotype	Vaccine effectiveness (95% CI) based on estimated serotype-specific threshold			Vaccine effectiveness based on 0.35 µg/mL correlate of protection		
	United Kingdom	Australia	Germany	United Kingdom	Australia	Germany
4	97% (61–100)	87% (59–97)	35% (2–97)	98%	99%	99%
6B	86% (3–100)	70% (15–94)	94% (61–100)	44%	95%	78%
9 V	75% (1–98)	60% (26–82)	80% (1–99)	86%	95%	98%
14	97% (86–100)	86% (26–99)	97% (68–100)	99%	100%	99%
18 C	99% (86–100)	80% (11–98)	74% (3–97)	90%	98%	98%
19 F	91% (50–99)	12% (2–89)	56% (4–91)	100%	100%	96%
23 F	87% (22–99)	67% (3–93)	88% (6–99)	72%	84%	87%
Aggregate	93% (20–100)	71% (3–97)	91% (5–100)	97%	98%	95%
Reported aggregate VE^a^	90% (34–98)^b^	70% (−8–92)^c^	90% (57–98)^d^	90% (34–98)^b^	70% (−8–92)^c^	90% (57–98)^d^

*CI*, confidence interval, *VE*, vaccine effectiveness.

^a^The values in the first three columns are repeated for ease of comparison.

^b^Data are from Andrews et al.^10^.

^c^Data are from Jayasinghe et al.^11^.

^d^Data are from Weinberger et al.^12^.

Data from Tables 2 and 3 are included here for ease of comparison.

## Discussion

With the widespread use of effective PCVs in the pediatric population, it is no longer feasible or ethical to perform placebo-controlled clinical efficacy studies for a new vaccine against IPD. Thus, current and future trials will continue to measure only the immune titers induced by a new vaccine. Therefore, evaluation of the potential impact of new PCVs on public health requires a method by which real-world effectiveness data can be reliably predicted from the immunogenicity data. As next-generation PCVs are developed in the coming years, this capability will be increasingly important in order to contextualize differences in immunogenicity between vaccines in terms of expected impact on public health. These modeled estimates provide a bridge between immunogenicity data and VE but should be used with caution and need to be verified with post-licensure evaluations of VE.

The model presented here enables serotype-specific estimates for C_p_ and for VE values, thus allowing predictions for and comparisons between current and future vaccines. The current standard practice is to use the aggregate C_p_ value of 0.35 μg/mL, derived for PCV7 serotypes, to predict and compare VE for not only the original seven serotypes, but also additional serotypes whose efficacy has not been shown in trials. Andrews et al. shows that the aggregate value is an imprecise predictor of the probable effectiveness of individual serotypes^10^. Performance of predicted VE was also evaluated: estimation of serotype-specific C_p_ results in better alignment between estimated and reported aggregate (incidence rate-weighted) VE (Table 5) compared to using the 0.35 μg/mL aggregate, as also suggested by Andrews et al.^10^.

Therefore, the serotype-specific estimates for C_p_ and VE obtained using the method described here provides a more accurate prediction of the probable protection afforded by PCV13 for the serotypes in common with PCV7. This modeling method has the potential to better estimate the effectiveness of next-generation PCVs against the serotypes shared with the current PCV, and, thus, to better inform public health decisions.

Several limitations should be kept in mind about the derivation of C_p_ and effectiveness as described here. The C_p_ and effectiveness prediction applies only to the prevention of IPD in children who resemble the trial populations. The lack of generalizability to other populations is because effectiveness is not only dependent on the strength of the immune response the vaccine elicits, but also on other factors^13^ including age at vaccination and the time interval between vaccination and serum sampling, which were found to explain 17–20% of the variance in antibody response to the serotypes in PCV7 and PCV13^14^. Geographic differences in the immune response to each serotype are also evident, with higher responses in children from South Africa than children living in the United States^9^. Such differences could have both genetic and environmental components, and is likely to depend also on dosing schedule and PCV valency^15,16^. Previous exposure to the serotypes could also be substantially different between countries and even within sub-populations, which may impact vaccine response and partial protection in the unvaccinated populations, resulting in a relative shift in effectiveness which could also change dynamically with fluctuations in relative incidence rates of circulating serotypes.

In addition to these considerations, several underlying assumptions also place limitations on the real-world applicability of our method. The effectiveness prediction does not use a functional assay output, like the pneumococcal opsonophagocytic killing assay, but instead relies on IgG concentration. However, previous work (Siber et al.^9^) demonstrates the utility of IgG in pediatric populations, thus mitigating any implied risk. Furthermore, opsonophagocytic killing assay titer values were not used in this work due to the relatively large assay variability (both between laboratories and over time) without a standard comparator assay, like the WHO ELISA for IgG concentrations, which is primarily used for licensure decision and to which concordance can be calculated for titer value normalization. The effectiveness is also the result of both uptake (percent of individuals vaccinated) and efficacy (relative risk reduction in a 100% vaccinated group relative to placebo recipients, randomized from an appropriately representative population). It can further depend on resulting secondary effects that include the reduced force of infection such as through “herd immunity,” and on changes over time in vaccine uptake or relative prevalence of serotypes (and concomitant changes in cross-protection). Additionally, this method assumes that equivalent antibody concentrations elicited by different PCVs (e.g., PCV7, PCV10, PCV13) for a specific serotype yield equivalent levels of protection against disease caused by that serotype. Current data across different manufacturers suggest this is a reasonable assumption (i.e., it is consistent with available data) but it needs to be verified with post-licensure VE studies.

In addition to methodological limitations, there were data limitations. One-month post-primary infant placebo titer concentrations were unavailable from the United Kingdom, Australia, and Germany, as no efficacy study was run in these countries. Placebo data were instead used from a PCV7 trial done in the United States, as the population in this study was assumed to have infants, which elicit placebo immune responses that closely mirror placebo immune responses in the United Kingdom, Australia, and Germany. One-month post-primary vaccination for subjects given either PCV7 or PCV13 in a 3 + 0 regimen was also unavailable in the Australian population. Trials from the United States were used here because the primary infant series dosing regimen of 2, 4, and 6 months is the same as the Australian primary infant series dosing regimen represented in the 3 + 0 regimen, and the populations are assumed to have similar immune responses (IgG concentrations) to PCV7 and PCV13. Last, effectiveness needed to be used rather than efficacy, as randomized controlled trials were not run for the vaccines, regions, and time periods of interest.

The method described here can be used to calculate the serotype-specific protective concentrations of antibodies elicited by PCVs, as well their serotype-specific effectiveness. To qualify the method, we applied it to calculate protective concentrations and effectiveness of PCV13 in three different geographic locations (United Kingdom, Australia, and Germany) using each country’s respective PCV7 serotype-specific effectiveness as input, as well as immunogenicity data that reflected the dosing regimen used to estimate the PCV7 effectiveness. No serotype-specific effectiveness has been reported for PCV13 against PCV7 serotypes (4, 6B, 9 V, 14, 18 C, 19 F, and 23 F), but aggregate effectiveness was reported against these seven serotypes for PCV13, and this aggregate was compared to the predictions. The serotype-specific predictions were aggregated (weighting by relative incidence rates) and the aggregated results agreed with the previously reported data, qualifying the method.

Using currently available population-level data, the method can predict serotype-specific effectiveness in next-generation PCVs. As next-generation PCVs are developed in the coming years, it will be important to estimate the shared serotype-specific effectiveness to contextualize differences in immunogenicity between vaccines in terms of expected effectiveness and identify whether next-generation vaccines will maintain (or, possibly, improve) control of serotypes that are currently controlled well. The serotype-specific effectiveness predictions may also be useful in dynamic transmission modeling to assess the potential of breakthrough disease, especially in higher-risk persistent serotypes (e.g., 3 and 19 A in Europe^3,17^).

## Methods

### Study design and data sources

**Fig. 1:**
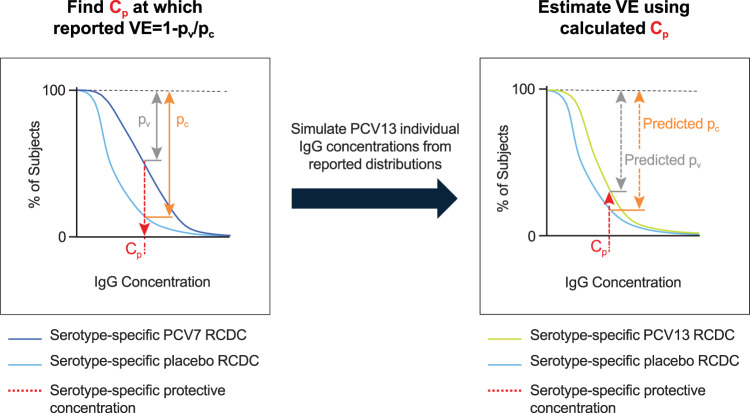
Modeling flow chart. The flow chart illustrates the two-step method for predicting the vaccine effectiveness (VE) of the PCV7 serotypes in PCV13. Beginning with the known serotype-specific IgG concentrations after vaccination with PCV7 (both placebo and active vaccine), simulated reverse cumulative distribution curves (RCDCs) are used, along with the known serotype-specific VE of PCV7 (where VE ≈ 1- (p_v_/p_c_)), (p_v_ is the percentage of subjects with antibody levels less than protective antibody concentration (C_p_) in the vaccinated cohort, and p_c_ is the percentage of subjects with antibody levels less than C_p_ in the control cohort) to derive the C_p_ that makes 1-(p_v_/p_c_) agree with reported VE for each serotype in PCV7 (red arrow in the left panel). Then, RCDCs are simulated for the PCV7 serotypes using PCV13 recipients’ serotype-specific PCV immunogenicity data. The C_p_ values previously derived for these serotypes are used to estimate the VE for those serotypes (see arrows in right panel).

The two-step method is illustrated in Fig. 1. Step 1 is based on the method described by Siber et al.^9^ for calculating the protective antibody concentration C_p_ when vaccine efficacy/effectiveness is known. Step 2 involves the calculation of VE based on C_p_. The method relies on previously reported serotype-specific values of VE for PCV7, and the concentration of antibodies raised against each of the PCV7 serotypes across placebo, PCV7, and PCV13 treated subjects one-month post-primary series. The one-month post-primary series was chosen as it (was used to derive and) is the timepoint used with the current correlate of protection (0.35 µg/mL) timepoint and reflects the immune response elicited within the first year of life when children are at the highest risk of IPD. The immunogenicity data are obtained from publicly available summaries of clinical trials, while the VE data are drawn from real-world evaluations of the vaccines (Table 4, and Supplemental Tables 1 and 2). PCV7 VE data were available from the United Kingdom^10^, Australia^11^, and Germany^18^. Immunogenicity data for PCV7 and PCV13 associated with the dosing regimens for the VE data inputs were obtained from the United Kingdom^19,20^ and Germany^21^. Australian 3 + 0 dosing regimen immunogenicity data for PCV7 and PCV13 were from a United States pediatric population^9,22^. Placebo data were also from a United States pediatric population^9^. The method was accordingly applied to data from the United Kingdom, Australia, and Germany.

VE data used for comparison to the predicted PCV13 VE are drawn from real-world evaluations of PCV13 in each country^10–12^.

### Derivation of the serotype-specific protective antibody concentration

In step 1, the known serotype-specific IgG concentrations after vaccination with PCV7 (both placebo and vaccine; Table 4) are used to simulate RCDCs, which plot the percentage of subjects at, or above, a given IgG concentration (Fig. 1). As subject-level IgG concentration data for placebo and PCV7 from the infant trials in the United Kingdom, Australia, and Germany were not available, individual IgG concentrations for placebo, PCV7, and PCV13 were simulated from summary-level published data (Table 4). The one-month post-primary series (for which the definition is country-specific) GMC and geometric standard deviation (GSD) for each serotype were used to simulate individual IgG concentrations from a log-normal distribution. The number of subjects simulated was based on the number of subjects in the respective vaccine treatment arm.

In addition to the serotype-specific IgG concentrations, Step 1 requires serotype-specific VE (Table 4) as input. As in Siber et al., VE is related to antibody concentration by the approximation:

1 $${{{\mathrm{VE}}}} \approx 1-\left( {{{{\mathrm{p}}}}_{{{\mathrm{v}}}}/{{{\mathrm{p}}}}_{{{\mathrm{c}}}}} \right),$$

where p_v_ is the percentage of subjects with antibody levels less than C_p_ in the vaccinated cohort, p_c_ is the percentage of subjects with antibody levels less than C_p_ in the control cohort, and C_p_ is the protective antibody concentration in µg/mL^9^. In this work, Eq. 1 uses the placebo and vaccinated RCDCs to give an estimate of VE at every IgG concentration C. C_p_ is the IgG concentration C at which that estimated VE is equal to the known (published) VE.

A number of simplifying assumptions were incorporated into step 1. The results assume that once the effects of antibody concentration are accounted for (by the protection model), the resulting efficacy and effectiveness are no longer dependent on the vaccination regimen (also known as “conditional independence”)^23^. They further assume that the relationship of the immune response and the probability of IPD is a step function (i.e., the probability of IPD is zero in subjects with serum antibody ≥C_p_), that the antibody concentration measured ∼4 weeks after the primary series immunization of infants predicts up to 5-year effectiveness, and that the reported serotype-specific GMCs and variance from the trials for placebo reflect the true population GMC and variance. It was also assumed that the proportions of subjects who missed vaccine doses were similar between cases and controls in the PCV7 VE publications and that the distribution of IgG concentrations from the study was representative of typical antibody concentration ∼4 weeks after the primary immunization, regardless of adherence. Finally, the reported PCV7 and PCV13 serotype-specific VE values were obtained during an observation period ranging from 2000–2010 (for PCV7) and 2010–2016 (for PCV13). An assumption was made that VE and force of infection remained unchanged across serotypes over those periods. It was further assumed that vaccine efficacy and the value of the C_p_ did not change (e.g., due to serotype replacement, change of circulating types giving cross-protection, etc.).

### Estimation of serotype-specific vaccine effectiveness

In step 2 (Fig. 1), Eq. 1 is applied to predict the VE of PCV13 for each serotype in common with PCV7. RCDCs are simulated for these serotypes based on the reported serotype-specific IgG concentrations in PCV13 recipients (active vaccine and placebo; Table 4). The C_p_ previously derived for these serotypes are then used to predict (using Eq. 1) the VE values for those serotypes. Here, it was assumed that IgG concentration is the only factor impacting VE for next-generation PCVs.

### Statistical procedures and assessment of predicted vaccine effectiveness

For each serotype/vaccine combination, the published GMC, the published VE, and their GSDs (calculated from the 95% confidence intervals) were used as inputs for Monte Carlo simulation (10,000 iterations). Each simulation sampled a new GMC and VE from a log-normal distribution; uncertainty in the GSD was accounted for by generating GSD estimates assuming a chi-square distribution.

Each simulation first solved for a serotype-specific C_p_ based on the RCDCs for placebo and PCV7 and the published VE values for PCV7 (step 1). We then solved for the serotype-specific VE values of PCV13 based on the C_p_ from step 1 and the simulated RCDCs for placebo and PCV13 (step 2). Simulated C_p_ (µg/mL) and VE (%) were summarized as medians with 95% confidence intervals.

Because only aggregate (not serotype-specific) values of VE were available in the literature for qualification of our results, the serotype-specific values calculated by our method were combined with weighting into aggregate medians, 2.5%, and 97.5% bounds. Weighting was based on the relative incidence rate of IPD caused by each serotype in PCV7, obtained from the source publications on VE^10,11,18^. The predicted VE values and confidence intervals were compared with the published values and confidence intervals.

These analyses were performed in the R statistical software version 4.1.0.

## Supplementary information

Supplementary Materials

## Data Availability

All input data are available in the tables.

## Code availability

The software used here can be found at this link: https://github.com/jryman17/pneuve.
