# Supplementary material for: Predicting vaccine effectiveness against invasive pneumococcal disease in children using immunogenicity data
Source: NPJ Vaccines. 2022 Nov 7;7:140. doi: 10.1038/s41541-022-00538-1 (PMC9640717; doi:10.1038/s41541-022-00538-1)
Supplement: Supplementary file 1 — Supplementary Materials [file 41541_2022_538_MOESM1_ESM.docx]

**Supplemental Table 1 Reported vaccine effectiveness study summaries for both model input (PCV7) and prediction comparison (PCV13)**

| VE estimate method | Vaccination regimen | IPD cases | Age group | Location | Source |
| --- | --- | --- | --- | --- | --- |
| PCV7 |  |  |  |  |  |
| Indirect cohort | 2+1 | 694 | 4-59 months | UK | Andrews et al.[[10](#_ENREF_10)] |
| Case-control | 3+0 | 1,209 | ≤48 months | Australia | Jayasinghe et al.[[11](#_ENREF_11)] |
| Indirect cohort | 3+1 | 46 | 5-24months | Germany | van der Linden et al.[[18](#_ENREF_18)] |
| PCV13 |  |  |  |  |  |
| Indirect cohort | 2+1 | 12 | 4-56 months | UK | Andrews et al.[[10](#_ENREF_10)] |
| Case-control | 3+0 | 308 | ≤42 months | Australia | Jayasinghe et al.[[11](#_ENREF_11)] |
| Indirect cohort | 3+1 | 8 | 4-56 months | Germany | Weinberger et al.[[12](#_ENREF_12)] |

UK, United Kingdom

**Supplemental Table 2 Reported summary-level IgG GMC study summaries used as model input^a^**

| Vaccination regimen | Number of subjects | Sampling point | Location of study | Source |
| --- | --- | --- | --- | --- |
| Placebo |  |  |  |  |
| 3+1 | 189 | 30 days post 3rd dose | US | Siber et al.[[9](#_ENREF_9)] |
| PCV7 |  |  |  |  |
| 2+1 | 123 | 30 days post 2nd dose | UK | Findlow et al.[[19](#_ENREF_19)] |
| 3+1 | 190 | 30 days post 3rd dose | US | Siber et al.[[9](#_ENREF_9)] |
| 3+1 | 279 | 30 days post 3rd dose | Germany | NCT00366340[[21](#_ENREF_21)] |
| PCV13 |  |  |  |  |
| 2+1 | 233-235^b^ | 30 days post 2nd dose | UK | Ladhani et al.[[20](#_ENREF_20)] |
| 3+1 | 387-399^b^ | 30 days post 3rd dose | US | NCT00444457[[22](#_ENREF_22)] |
| 3+1 | 285 | 30 days post 3rd dose | Germany | NCT00366340[[21](#_ENREF_21)] |

UK, United Kingdom

^a^Summarized using WISSPAR: A curated immunogenicity database with public facing dashboards. Available from: <https://wisspar.com>.

^b^Number of subjects with measured IgG concentrations varied across serotypes evaluated.
